# Oncolytic Virus-Induced Autophagy in Glioblastoma

**DOI:** 10.3390/cancers13143482

**Published:** 2021-07-12

**Authors:** Margarita Kamynina, Salome Tskhovrebova, Jawad Fares, Peter Timashev, Anastasia Laevskaya, Ilya Ulasov

**Affiliations:** 1 Group of Experimental Biotherapy and Diagnostic, Institute for Regenerative Medicine, Sechenov First Moscow State Medical University, 119991 Moscow, Russia; margaret.kamynina@gmail.com (M.K.); sali.bibikc@gmail.com (S.T.); ayaksveal@yandex.ru (A.L.); 2 Department of Neurological Surgery, Feinberg School of Medicine, Northwestern University, Chicago, IL 60611, USA; jawad.fares@northwestern.edu; 3 Institute for Regenerative Medicine, Sechenov First Moscow State Medical University, 119991 Moscow, Russia; timashev_p_s@staff.sechenov.ru; 4 Department of Polymers and Composites, N. N. Semenov Institute of Chemical Physics, 119991 Moscow, Russia; 5 Chemistry Department, Lomonosov Moscow State University, 119991 Moscow, Russia

**Keywords:** autophagy, oncolytic virus, apoptosis

## Abstract

**Simple Summary:**

Glioblastoma (GBM) is the most common and aggressive brain tumor with an incidence rate of nearly 3.19/100,000. Current therapeutic options fall short in improving the survival of patients with GBM. Various genetic and microenvironmental factors contribute to GBM progression and resistance to therapy. The development of gene therapies using self-replicating oncolytic viruses can advance GBM treatment. Due to GBM heterogeneity, oncolytic viruses have been genetically modified to improve the antiglioma effect in vitro and in vivo. Oncolytic viruses can activate autophagy signaling in GBM upon tumoral infection. Autophagy can be cytoprotective, whereby the GBM cells catabolize damaged organelles to accommodate to virus-induced stress, or cytotoxic, whereby it leads to the destruction of GBM cells. Understanding the molecular mechanisms that control oncolytic virus-induced autophagic signaling in GBM can fuel further development of novel and more effective genetic vectors.

**Abstract:**

Autophagy is a catabolic process that allows cells to scavenge damaged organelles and produces energy to maintain cellular homeostasis. It is also an effective defense method for cells, which allows them to identify an internalized pathogen and destroy it through the fusion of the autophagosome and lysosomes. Recent reports have demonstrated that various chemotherapeutic agents and viral gene therapeutic vehicles provide therapeutic advantages for patients with glioblastoma as monotherapy or in combination with standards of care. Despite nonstop efforts to develop effective antiglioma therapeutics, tumor-induced autophagy in some studies manifests tumor resistance and glioma progression. Here, we explore the functional link between autophagy regulation mediated by oncolytic viruses and discuss how intracellular interactions control autophagic signaling in glioblastoma. Autophagy induced by oncolytic viruses plays a dual role in cell death and survival. On the one hand, autophagy stimulation has mostly led to an increase in cytotoxicity mediated by the oncolytic virus, but, on the other hand, autophagy is also activated as a cell defense mechanism against intracellular pathogens and modulates antiviral activity through the induction of ER stress and unfolded protein response (UPR) signaling. Despite the fact that the moment of switch between autophagic prosurvival and prodeath modes remains to be known, in the context of oncolytic virotherapy, cytotoxic autophagy is a crucial mechanism of cancer cell death.

## 1. Introduction

Glioblastoma (GBM) is the most common lethal primary brain tumor among adults. Survival outcomes continue to be dismal despite aggressive therapeutic regimens that include surgical resection, chemotherapy, and radiation [1]. The median survival of patients does not exceed 20 months [2]. Therefore, the search for or development of effective antiglioma strategies is necessary to achieve a breakthrough in the current status quo. Most recently, anticancer therapy with oncolytic viruses showed a strong antiglioma effect [3] and became a viable option in clinics [4].

Oncolytic viruses are a vast group of agents that utilize the replication cycle of a host to deliver and express toxic viral proteins upon infection of various cancer cells [3]. Depending on the viral antigen structure and the virion’s platform, oncolytic viruses may cause a characteristic inflammatory response [5,6], act as an adjuvant therapeutic [7,8], serve as a vaccine agent in situ [9,10], function as an anticancer agent for systemic injection [11,12], and/or cause DNA damage in cancer cells [13]. Furthermore, oncolytic viruses can lead to multiple types of programmed cell death [6,14,15,16] since targeted genes are involved in the cell signaling of apoptosis, pyroptosis, necroptosis, and autophagy. 

Autophagy is a process that is characterized by the development of a double-membraned phagosome in response to cellular stress [17]. Autophagy is known to play a dual role in cell survival, both cytoprotective and cytotoxic. Under normal physiological conditions, autophagy is required to get rid of damaged organelles and mitochondria and to provide roadblocks for protein synthesis and energy storage [18]. During cancer progression, autophagy is used by the tumor to maintain the pathological state of the tumor cells [19]. In the context of cytotoxicity, the autophagic type of cell death resembles the process of cellular self-consumption due to stress the cell is unable to cope with. Autophagy induction is important for enhancing the cytotoxic effect mediated by the Newcastle disease virus [20] and other oncolytic viruses that are interferon-β-armed [16] or based on Delta24RGD [21]. Various oncolytic viruses have been studied in clinical and preclinical studies. Adenovirus, blueberry virus, herpes virus, parvovirus, picornavirus, and reovirus were investigated in human and veterenary medicine [22]. On clinicaltrials.gov (accessed on 20 April 2021), 20 trials have explored oncolytic viruses for GBM treatment. The most studied were Vaccinia virus, adenovirus, Newcastle Disease, and HSV.

**Figure 1:**
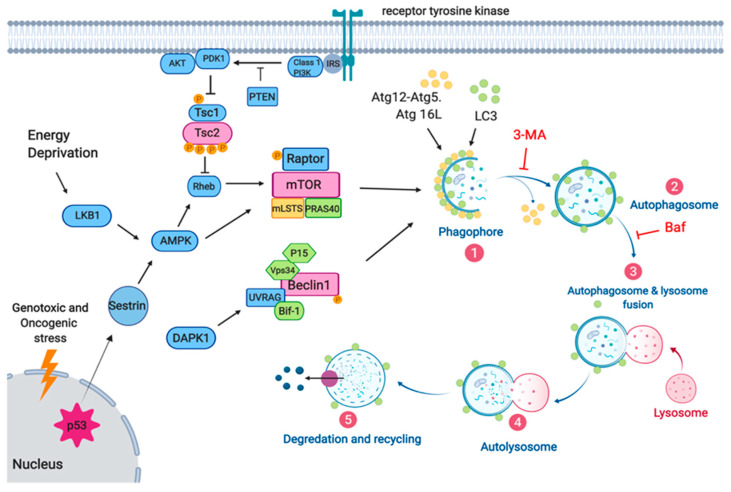
Autophagy regulation pathways in response to stress signal. The figure shows the pathway for the initiation of autophagy through the activation of a receptor tyrosine kinase. The autophagy process includes initiation, elongation, autophagosome formation, lysosome fusion, and degradation. The initiator complex is a base for launching the assembly of the autophagosomal membrane de novo. It consists of Beclin-1, Bcl-2, VPS (vacuolar protein sorting) kinase-34, and Atg14L proteins. The Atg5-Atg12 complex interacts with Atg16L1 and then participates in the elongation of the autophagosomal membrane. Atg4B cleaves LC3I to LC3II, which, after conjugation with PE (phosphatidylethanolamine), turns into an autophagosomal membrane-bound protein directly involved in membrane formation. After fusion, all membrane proteins (except LC3II) involved in this process leave the autophagosome.

Concerning the molecular basis, the main molecular regulators of autophagy in cancer are the LKB1–AMPK pathway, the PI3K/AKT pathway (via mTOR), Beclin 1, and p53 [23]. The autophagy regulation pathways in response to stress signals are shown in Figure 1. The cellular response to stress induced by an oncolytic virus—for example, CRAd-S-pK7—involves the initiation and maintenance of intracellular signals using reactive oxygen species (ROS) [24], lipid products [25], and/or other signal transmitters. The modulation of these factors impacts the initiation of cellular signaling that is necessary for cell death and thus provides a challenge for anticancer approaches. Here, in this review, we explore the cellular and molecular events involved in the induction of cytoprotective autophagy-mediated responses to oncolytic viruses in glioblastoma, which reduces oncolytic efficacy, and mechanisms of cytoprotective-cytotoxic autophagy switch, which is possibly capable of improving the oncolytic effect.

## 2. Autophagy in Cancer Cells as Both a Defense Mechanism Against Viruses and the Initiating Cell Death Process

Autophagy signaling is vital to fight pathogens such as bacteria or viruses, but in transformed cells, autophagy signaling is weakened due to the inherent immunosuppressive properties of cancer cells [26,27]. As such, the response of tumor cells to viral infection by OVs is often diminished, despite partial responses witnessed against viral antigens. The differences in cellular responses triggered cancer biologists to explore the mechanisms of action that govern the OV-induced response.

Recent investigations indicate that the integral role of autophagy signaling in cancer cells is associated with stress resistance, hinting at the biological significance of autophagy in cell survival [28]. Therefore, autophagy helps cancer cells maintain cellular integrity after viral infection and permits cancer cells to strike a balance between the need to react to stressful stimuli and cell survival and protection. This established a paradigm whereby viral agents and antigens can be distinguished based on the cellular response and stress magnitude induced in cancer cells. In other words, released stress factors require autophagic induction as a response to the internalized viral agents in the cytoplasm to promote cancer cell adaptation and survival [29].

As already stated, oncolytic viruses induce autophagy, which results in cancer cell lysis; hence, OV agents may switch the cytoprotective role of autophagy to cytotoxic. Oncolytic viruses are considered a therapeutic option in neuro-oncology in general and glioblastoma in particular, whereby OVs are required to internalize into target cells and induce cellular death, affecting overall tumor proliferation. In the following sections, we highlight the cellular reactions mediated by oncolytic viruses.

## 3. Regulation of Apoptotic Proteins Provides Advantages for Oncolytic Virus-Mediated Cell Death

Apoptosis is a key cellular mechanism that protects cells against viral infections [30]. Together with selective autophagy, apoptosis is an element of the protective immunity of the cell that eliminates microbes and enhances immune recognition of antigens expressed on cell surfaces. Similarly, endoplasmic reticulum stress (ER stress), which is followed by the unfolded protein response (UPR); apoptosis; and autophagy are signaling elements of neuroprotective protein unfolding during viral infection of the central neural system (CNS) [29].

Apoptosis as a prosurvival and/or prodeath process has been explored in glioblastoma cells treated with chemotherapeutic drugs or oncolytic viruses. Moreover, accumulating evidence [31,32] suggests some correlation between glioblastoma resistance and the activity of apoptotic proteins. To date, the apoptotic pathways through which oncolytic viruses, such as M011L-oncolytic myxoma virus, CRAd-S-5/3, and Delta24RGD, kill glioma cells are not fully understood, and present data suggest a complex contribution of Bcl-2-like protein 4 (BAX) [33], p53 upregulated modulator of apoptosis protein (PUMA) [13], and B-cell lymphoma 2 protein (BCL2) [21,33] in tumor inhibition. Most recently, it was shown that the inhibition of Bcl2 potentiates cancer cells to the M1 vector via upregulation of BCL2 antagonist/Killer 1 (Bak) [25]. Previously, it was reported that primary GBM samples are upregulated with survivin (Birc5) [34] and BCL2 [35], preventing the induction of BAX, BID, or BAK upon oncolytic virus infection through the expression of inhibitory molecules for apoptosis. Therefore, apoptosis induction during oncolytic virus infection results in BAX-independent cell death by promoting durable cellular stress. Thus, revealing the mechanism of regulation of apoptotic proteins will allow better stimulation of OV-mediated cell death.

## 4. The Link between Apoptosis and Autophagy: ER Stress

The prolonged stress response via the expression of proapoptotic proteins may result in a disturbance in the endoplasmic reticulum (ER) environment as unprocessed proteins accumulate in the ER. This disproportion with the expression of antiapoptotic proteins such as BCL2 and BCLXL governs cellular reactions and causes durable stress [36]. In cells such as glioma ER stress recruits elements of the tumor microenvironment [37] to enhance tumor progression and expansion via ER signaling. This includes inositol-requiring enzyme 1α (IRE1α), activating transcription factor 6 (ATF6), and pancreatic ER kinase-like ER kinase (PERK) localized in the ER. Additionally, IRE1α acts as ER stress sensor [38]. Interestingly, both PERK and ATF1 are inducers of autophagy, while IRE1α is its negative regulator. Moreover, the lower the level of IRE1α expression in glioblastoma cells, the higher the therapeutic effectiveness of the oncolytic virus M1 [39]. Emerging evidence suggests that X-box binding protein (XBP1), an IRE1α downsignaling, and calreticulin, an ER-resident chaperone that has been localized to the surface of tumor cells [40], are both linked to glioma progression [38]. Tumors exhibiting high IRE1 α activity also correlated with bad outcomes and shorter median survival in patients. Therefore, the accumulation of unprocessed proteins in the ER due to apoptotic protein expression produces ER stress. ER stress in turn promotes tumor progression. However, at the same time, ER stress is linked to autophagy activation, and a low level of IRE1 alpha, a negative regulator of autophagy, contributes to the higher effectiveness of antiglioma agents. Thus, ER stress plays a dual role in tumor progression. It recruits elements of the tumor microenvironment to enhance tumor progression but also improves the therapeutic efficacy of the oncolytic virus M1 through inducing autophagy [39].

## 5. Unfolded Protein Response (UPR): One Process, Two Impacts

The unfolded protein response is a process activated due to overwhelming ER stress and the inability of tumor cells to counteract it. It remains unknown at which point the UPR leads to cell death, how it promotes cellular apoptosis, and/or when it promotes cell survival [41]. UPR has long been viewed as an adaptive program to sustain cellular homeostasis to counterbalance ER stress. ER stress is often developed as a secondary mechanism to drug treatment or viral persistence. This is the case with HCMV [42], the hepatitis C virus [43], and the hepatitis B virus [44]. The infection of normal brain cells [45,46] and/or glioma cells [47,48] with retroviruses, NDV, or MDA-7/IL-24 also leads to ER modifications in cellular programming. Treatment regimens and viral proteins may put the ER under stress, but vulnerable cells cope with stress-induced stimuli. However, if failure in eliminating the source of cellular stress occurs and extensive ER stress is unable to restore ER balance, the activated UPR aims to eliminate the damaged cells and promote cell death. Therefore, UPR-modulating drugs and approaches have emerged as promising candidates for mono- and combination therapy with temozolomide against glioblastoma.

When UPR fails to rebuild tumor homeostasis, autophagy induction occurs. Autophagy is a compensatory mechanism to eliminate the burden of toxic proteins in the ER. Damaged organelles can be subjected to destruction inside autophagosomes. Interestingly, a study completed by Mahoney et al. [49] described the mechanism of UPR inhibition caused by an oncolytic rhabdovirus that improved its cytotoxicity against adherent and primary patient-derived glioblastoma cells. Growing evidence suggests that the fitness and fate of tumor cells infected with oncolytic viruses can be the result of countermovement between UPR and autophagy. Li et al. were the first to demonstrate a link between autophagy induction and tumor resistance upon infection with an oncolytic virus [39]. In this study, autophagy was shown to follow UPR. In particular, infection with oncolytic M1-alphavirus in U87 glioma cells triggers UPR and subsequent autophagy, while blocking UPR and autophagy resulted in enhanced antitumor efficacy of the vector. Since then, various attempts have been made to explain the response mechanism that governs glioma protection against infection. It turned out that M1 activates UPR in primary malignant glioma cells via binding of an incorrectly folded protein to the ER chaperone Bip / GRP78. This is followed by the release of three membrane-bound proteins: IRE1, PERK, and ATF6. Currently, it is understood that UPR is regulated by membrane-incorporated sensors including double-stranded RNA-activated protein kinase (PKR)-like ER kinase (PERK), activating transcription factor 6 (ATF6), and inositol-requiring enzyme 1 (IRE1). Thus, in the context of molecular basis, while PERK and ATF6 are responsible for the induction of autophagy, IRE1 exhibits dualism in autophagy regulation. Additionally, activation of PERK-eIF2a-ATF4 affected the activity of Beclin 1, ATG16L1, and p62 [50], suggesting a link between UPR and autophagy modulation. Meanwhile, autophagy and thus UPR are shown to play a prosurvival role.

On the molecular level, the variability of the UPR has been studied by modulating the level of IRE1a expression in glioblastoma cells. Blocking IRE1a leads to the inhibition of the expanded protein response, which is sufficient for the virions’ assembly and their release from the infected cells. At the same time, the increase in viral spread by stimulating the UPR is one of the approaches to promote tumor cell killing. Prasad et al. demonstrated that the UPR was greatly enhanced by the simultaneous treatment of tumor cells with adenovirus and the inhibitor of Golgi-specific brefeldin A-resistant guanine nucleotide exchange factor 1 (GBF1) [51]. Moreover, the boost of oncolytic virus titer and the increase in cytopathic effect were attributed to tumor cells, suggesting a dependence of HAdV-C5 or HAdV-B on the UPR response. Considering all the above, since most anticancer studies use the HAdV-C5 as a vector to deliver a therapeutic payload to the target cells, stimulation of UPR, activation of autophagy, and progeny release become paramount tasks that affect oncolytic virus spreading. This rule can be applied to other oncolytic viruses such as mouse cytomegalovirus (MCMV) [50] and Kaposi-sarcoma-associated herpesvirus (KSHV) [52], which can infect and persist in neural cells. Another example of ER stress and autophagy activation is the infection of glioma cells with Chikungunya (CHIKV) alphavirus [53]. In vitro experiments showed that the oncolytic virus required autophagy induction for viral replication. A similar effect was seen with an adenovirus that uses Golgi and ER containers for virion release, suggesting a role for autophagy in antipathogen defense [54]. In this regard, we consider that compared to autophagy, apoptosis might be more devastating for glioma cells, since the lack of regenerative functions made neural stem cells vulnerable to destruction. Therefore, the UPR becomes an adaptive reaction of target cells to protect themselves from the accumulation of toxic proteins, including viral ones. In support of this hypothesis, during host cell infection, adenoviruses synthesize an excessive amount of hexon and fibers that are not used for new capsid assembly [55]. Additionally, it has been shown that Nef-protein, encoded by human immunodeficiency virus type 1 (HIV-1), blocks the fusion of the autophagosome with the lysosome, thereby keeping the virus inside cells [56]. Later, the virus found to encodes the protein Tat promote autophagy in neuronal cells [57] and gliomas via BAG3 signaling [57]. 

Overall, UPR plays a dual role in cancer cell survival. On the one hand, UPR is one of the pathways of autophagy induction and thus is capable of enhancing tumor resistance to infection with oncolytic vectors, but, on the other hand, stimulation of UPR promotes enhanced virions’ assembly and release, thus promoting cytotoxicity. Since various OVs, such as reolysin [58], virus M1 [39], HAdV-C2-dE3B_GFP [59], 34.5ENVE [60], and hTert-Ad [61], modulate or require UPR to shift the balance between cell defense and cytotoxicity, establishing a link between cell defense and the viral response mechanism has broader clinical connotations.

## 6. Cytotoxic Autophagy in Studies with Oncolytic Viruses

As it was demonstrated previously, phenotypical and genotypical changes acquired after therapy produce aggressive subtypes of GBM cells that are resistant to the standard of therapy [62,63]. Therefore, much effort has been made to understand the mechanism of GBM resistance and discover novel molecular targets and potential effective drugs to target this disease [32,33].

Infection of GBM cells with an oncolytic virus causes the production and accumulation of viral proteins in the cytoplasm and the accumulation of unfolded proteins in the ER. These conditions launch transcription and translation of proteins to restore the cellular “status quo”. While UPR aims to remove proteins from the ER and promote cell survival, the prolonged UPR also triggers autophagy via the induction of autophagy-related proteins [64].

**Figure 2:**
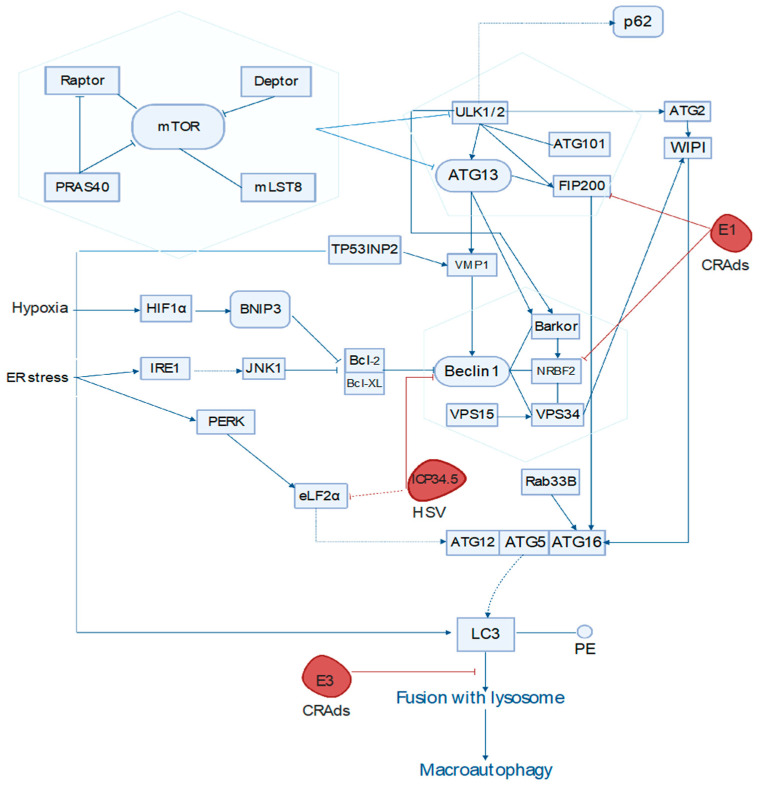
Influence of viral proteins (CRAds and HSV-1) on autophagy. Intermittent lines describe the indirect effect, and continuous lines describe the direct. Adenoviral E1 protein inhibits FIP200 and NRBF2 proteins in the autophagy pathway. Interaction of FIP200 and ATG16 defies ULK1-dependent or independent induction of autophagy. E3 protein prevents the fusion of lysosomes. ICP34.5 of HSV-1 inhibits autophagy, inhibiting eLF2α and Beclin1.

It is worth mentioning that various adenoviruses, such as OBP301 [65], delta24RGD [66], FAdV-4 [67], SG511-BECN [68], Ad.wnt-E1A(△24bp)-TSLC1 [69], and mda-7/IL-24 [70], require autophagy to induce programmed cell death [71]. Among all vectors, conditionally replicating adenoviruses (CRAds) are promising agents for the treatment of tumors because of their ability to multiply and cause lysis of tumor cells and their surroundings [72]. Over the decades, it has been shown that protease L3 and E3-11.6-kDa adenovirus death protein cause the reorganization of host skeleton and contribute to adenovirus spreading [73,74]. To avoid side effects and improve tropism for the tumor, the virus is genetically engineered with the insertion of the human telomerase reverse transcriptase promoter (hTERT), which affects the expression of the adenoviral E1A gene, allowing oncolytic virus replication only in tumor cells (telomerase-positive cells) [75,76]. E1A induces caspase-3-mediated apoptosis independent of p53 [77]. A study by Ito et al. showed that cytotoxic autophagy is mediated by the blockade of the mTOR signaling pathway after exposure to hTERT-Ad viruses of U373-MG and U87-MG cells in vivo and in vitro [75]. Whether these events are important for autophagy regulation remains to be investigated, but the deletion of protease ICP10K [78], known for binding with Beclin 1, from the genome of HSV2, another vastly used gene therapy vehicle, triggers cell-specific apoptosis [79] in cancer cells. The precise mechanism of viral proteins’ influence (CRAds and HSV-1) on autophagy is depicted in Figure 2.

In the context of other oncolytic vectors and autophagy activation, the herpes virus also contains the ICP34.5 protein significant for oncolytic virotherapy, which is responsible for binding to Beclin 1 at the subsequent inhibition of eIF2α. Additionally, Kanai et al. showed that the Δ68H-6 virus modified HSV-1 (with deletionγ34.5) is an effective vector for the treatment of glioblastoma since it is not able to inhibit autophagy [80]. Another example is flavivirus Zika that also restricts ER stress turnover for virus replication by cleavage of the FAM134B, an autophagy receptor that is responsible for targeted ER degradation [81]. Inhibition of intracellular ATP levels and IRE1α or PERK causes cytotoxic autophagy mediated by Newcastle disease virus (NDV) [82] and M1 [39] viruses, relatively. Thus, various oncolytic viruses are capable of inducing cytotoxic autophagy which promotes their oncolytic efficacy. 

Despite the general consesnus reached on the role of autophagy in oncolytic virus cytotoxicity, several studies reported a negative contribution of autophagy in CRAd-mediated cell death, suggesting the cytoprotective role of autophagy during virus infection. For instance, a study by Botta et al. [83] showed that dl922-947 triggered an autophagy response, whereas the inhibition of infected cells with 3-Ma increased the anticancer effect mediated by the viral vector. Bhutia et al. [83] further observed cytoprotective autophagy in the case of prostate cancer cell infection with Ad.*mda*-7 virus [84]. Although both viral groups used delta 24 adenovirus backbone, infected cells displayed different autophagic responses. 

## 7. Modulation of Cytotoxic Autophagy with Oncolytic Virus or Drug Combination

Overall, this review highlights how important it is to counteract autophagy mediated by infected cells. Despite clear evidence that OV modulates autophagy, the exact method of induction of cytotoxic autophagy and the cellular proteins involved remain unclear. Recent findings indicate that ATG proteome provides a cellular function outside autophagy signaling, which involves a host–pathogen interaction [85]. More than 20 ATG-based proteins have been shown to inhibit or support viral replication of several self-replicated vectors, such as adenoviruses (dl922-947 [83], delta24RGD [66,86], CRAd-S-pK7 [87]), adchikungunya virus [88], HSV(Δ68H-6 and 1716-6 [80]), or NDV (strain Beaudette C [20]). It appears that various vectors have preferences for autophagy protein expression and that such interaction suggests an interplay between autophagy-related proteins and viral replication cycles. This evidence may confirm previous findings that siRNA interference against ATG5 or Beclin1 suppresses oncolytic vector replication and progeny release in the case of NDV (strain Beaudette C [20]) or adenovirus-based CRAd-S-pK7 [87] and Adhz60 (deltaE1B) [89]. In addition, an association between herpes simplex virus-1 produced proteins [90,91] or enterovirus A71 [92] and autophagy-related proteins has recently been reported, further confirming the possibility of the nonautophagy canonical function of autophagy-related proteins during the replication of several oncolytic viruses. Thus, different oncolytic viruses are capable of inducing various forms of autophagy, including both cytotoxic and cytoprotective forms.

## 8. Conclusions

Cell death is a highly conserved process, the understanding of which is essential for the construction of experimental drugs, such as oncolytic viruses that have shown promising results in glioblastoma treatment. The effectiveness of therapy depends on the ability of the oncolytic virus to infect and spread in tumor cells. One of the main problems in the introduction of oncolytic virotherapy in clinics is an incomplete understanding of the mechanisms of action of the oncolytic virus on the tumor and its microenvironment. More specifically, this includes autophagy, which induces cell death and enhances virus particle spread through the modulation of UPR.

Cell death via oncolytic virus infection involves the recognition of viral particles by host cells followed by an attempt to destroy them via autophagy inside the autophagosomes. Accordingly, ER stress and UPR are modulators of the vesicular stomatitis virus [49] and herpes simplex virus [60] cytotoxicity during cellular response to infection. Moreover, proapoptotic protein expression seems to play an important role in this process. Despite a large number of studies in the field of autophagy and oncolytic viruses, it is still unclear what precedes the prosurvival to prodeath autophagy switch. In addition, it became known that the form of autophagy depends on the type of oncolytic vector. Therefore, it is important to understand the pathways that regulate autophagy signaling and determine ways to enhance the cytopathic effect mediated by oncolytic vectors [93].

## 9. Future Directions

Currently, the therapeutic effect of oncolytic viruses is achieved by combining different types of therapies: for example, oncolytic virotherapy and chemotherapy. It has recently been acknowledged that the activation of cellular autophagy can be greatly enhanced by the addition of autophagy-inducing drugs such as rapamycin [94,95]. It is known that rapamycin is an immunosuppressor that acts through the interaction between the 12-kDa FK506-binding protein (FKBP12), a rapamycin-associated cellular protein, and FKBP12 target 1 (FRAP/mTOR/RAFT1). It inhibits G cell cycle progression in mammalian cells [96]. Given the fact that various oncolytic viruses arrest cells in the S phase of the cell cycle, an additive effect between oncolytic viruses—for example, Myxoma virus [97,98], Newcastle virus [20], adenovirus OBP-405 [71], vaccinia virus JX-594 [99] and vvDD-EGFP [100], IFN-sensitive VSV-mutant strain (VSV(DeltaM51) [101], and rapamycin—may result in increased cytotoxicity. Strong additive effects can also be seen by the combination of oncolytic herpes simplex viruses and cell death inducers of apoptosis (etoposide) [102], the combinaiton of ionizing radiation with parvovirus M1 or myxoma virus [103,104,105], and the combination of inducers of cell cycle arrest and apoptosis with oncolytic viruses, such as Newcastle virus, vericular stomatitis virus, or vaccinia virus in temozolomide-treated glioblastoma cells [106,107]. Thus, a combination of oncolytic virotherapy with autophagy-stimulating agents seems to be a promising strategy for future clinical trials.

## Abbreviations

ATF6	activating transcription factor 6
ATG5 and ATG7	autophagy-related protein 5 and 7
BAK	BCL2 Antagonist/Killer 1
BAX	bcl-2-like protein 4
BID	pro-apoptotic Bcl-2 protein
BCL2	B-cell lymphoma 2 protein
BCLXL	B-cell lymphoma-extra-large
CMV	cytomegalovirus
CNS	central neural system
FKBP12	12-kDa FK506-binding protein
GBM	glioblastoma
IRE1α	inositol-requiring enzyme 1α
JNK	c-Jun N-Terminal Kinase
ER	endoplasmic reticulum
HadV-C5	human adenovirus, C type, strain 5
HDACi	histone deacetylase inhibitors
HIV-1	human immunodeficiency virus, type 1
HSV	herpes simplex virus
OV	oncolytic virus
PE	phosphatidylethanolamine
PERK	pancreatic ER kinase-like ER kinase
ROS	reactive oxygen species
UPR	unfolded protein response
VACV	vaccinia virus
VPS	vacuolar protein sorting
XBP1	X-box binding protein
